# Association Between Childhood Maltreatment, *FKBP5* Gene Methylation, and Anxiety Symptoms Among Chinese Adolescents: A Nested Case-Control Study

**DOI:** 10.3389/fpsyt.2022.761898

**Published:** 2022-02-03

**Authors:** Wenjian Lai, Wenyan Li, Xueying Du, Yangfeng Guo, Wanxin Wang, Lan Guo, Ciyong Lu

**Affiliations:** ^1^Department of Medical Statistics and Epidemiology, School of Public Health, Sun Yat-sen University, Guangzhou, China; ^2^Health Promotion Center for Primary and Secondary Schools of Guangzhou Municipality, Guangzhou, China

**Keywords:** childhood maltreatment, DNA methylation, anxiety symptoms, association, adolescents

## Abstract

**Background:**

Anxiety symptoms are common mental health problems among adolescents worldwide. This study aimed to explore (1) the longitudinal association between childhood maltreatment and anxiety symptoms, (2) the association between childhood maltreatment and DNA methylation of the *FKBP5* gene, and (3) the association of DNA methylation of the *FKBP5* gene with anxiety symptoms at follow-up.

**Methods:**

A nested case-control design was conducted to identify a case group and control group from a longitudinal study of adolescents aged 13–18 years in Guangzhou from 2019 to 2020. Adolescents with anxiety symptoms at baseline and follow-up were considered the case group, while those without anxiety symptoms at baseline and follow-up were considered the control group. The case and control groups were matched according to age and sex. Our study finally included 97 cases and 141 controls.

**Results:**

After adjusting for significant covariates, childhood emotional abuse was associated with subsequent anxiety symptoms (β = 0.146, 95% *CI* = 0.010~0.283); students with physical and emotional neglect were more likely to get a lower level of DNA methylation at most CpG units of *FKBP5* gene (*P* < 0.05); *FKBP5*-12 CpG 15 methylation was associated with anxiety symptoms at follow-up (β = −0.263, 95% *CI* = −0.458~-0.069). However, after multiple hypothesis testing, childhood maltreatment was not associated with *FKBP5* DNA methylation (*q* > 0.10); *FKBP5* DNA methylation did not show an association with subsequent anxiety symptoms (*q* > 0.10).

**Conclusions:**

Childhood emotional abuse was associated with an increased risk of anxiety symptoms among Chinese adolescents. After multiple hypothesis testing, childhood maltreatment was not significantly associated with *FKBP5* DNA methylation. DNA methylation of the promoter region of the *FKBP5* gene was not a significant predictor of anxiety symptoms. More attention should be paid to the mental health of adolescents with childhood maltreatment.

## Introduction

Anxiety symptoms are a common mental problem among adolescents worldwide (1). Exposure to anxiety symptoms in adolescence exerts a long-term negative impact on cognitive, behavioral, and social abilities (2, 3). Adolescents with anxiety symptoms are more likely to develop anxiety disorders in subsequent adulthood (4), imposing a heavy economic burden on families, and society due to treatment (5). Previous studies showed that 10–30% of adolescents reported anxiety symptoms globally (4, 6), and about 27.3% of Chinese middle school students suffered from anxiety symptoms (7). Although anxiety symptoms are multifactorial (1), compelling evidence has demonstrated that adverse life events, including childhood maltreatment, are significantly associated with anxiety symptoms (8, 9). Moreover, a history of childhood maltreatment increases an individual's vulnerability to the development of anxiety (10).

Childhood maltreatment includes physical, emotional, and sexual abuse as well as physical and emotional neglect. The experience of childhood maltreatment exerts a negative influence on the development of cognitive and emotional regulation and leads to dysfunction of the hypothalamus-pituitary-adrenal (HPA) axis (9, 11). HPA axis, which regulates the synthesis and release of glucocorticoids, is one of the key stress response systems. Once an individual is continuously exposed to an environmental stressor, it can cause changes in the ability of the HPA axis to respond to stress, even resulting in dysfunction (12, 13). The dysregulation in this system has been proved as a risk factor for stress-related psychiatric disorders, such as anxiety and depression (14). Besides, previous studies have indicated that adolescents exposed to childhood maltreatment are more likely to emerge anxiety symptoms (10, 15).

Substantial literature supports an association between adverse life events and poor mental health outcomes (15, 16). However, the molecular mechanism that accounts for such a solid and lasting effect has not been adequately studied. Evidence suggests that sustained exposure to adverse life events such as childhood maltreatment may lead to dysfunction of the stress response system. To adapt to such changes, the organism will undergo stable and lasting epigenetic modifications, such as DNA methylation, which can affect gene expression (13, 17). DNA methylation is an epigenetic modification that regulates gene expression without changing the DNA sequence. A growing body of studies indicates that abnormal DNA methylation is associated with a higher risk of stress-related mental disorders (18, 19). Additionally, altered methylation may affect the expression of genes related to the stress response system, suggesting that it plays a vital role in the pathogenesis of mental disorders (20). FK506 binding protein 5 (*FKBP5*) gene is an essential regulatory in the stress response system whose product is involved in the regulation of glucocorticoid receptor (GR) activity in the intracellular ultra-short negative feedback loop (21). Previous studies have shown that alteration in DNA methylation of the *FKBP5* gene is associated with mental disorders such as anxiety or depression (22, 23). Besides, evidence suggested that individuals with childhood maltreatment showed lower levels of methylation of the *FKBP5* gene compared to those without maltreatment (21, 24). Consequently, the above evidence provides some information that DNA methylation of the *FKBP5* gene may play an important role in the association between childhood maltreatment and anxiety symptoms.

We thus conducted this prospective study among Chinese adolescents to explore (1) the longitudinal association between childhood maltreatment and anxiety symptoms, (2) the association between childhood maltreatment and DNA methylation of the *FKBP5* gene, and (3) the association of DNA methylation of the *FKBP5* gene with anxiety symptoms at follow-up.

## Methods

### Study Design and Participants

The present study used a prospective nested case-control study design based on the Longitudinal Study of Adolescent's Mental and Behavioral Well-being in Guangzhou, China (25, 26) (Registration No. ChiCTR1900022032). The longitudinal study has been carried out in 10 public high schools from 4 main urban districts of Guangzhou, and students in grade 1 within the chosen schools were invited to participate voluntarily. At baseline (January to April 2019), a total of 1,956 students were recruited, standardized self-administered questionnaires were fulfilled, and a peripheral blood sample (5 ml) from participants was also collected. The follow-up survey was conducted at 1-year later. Students with anxiety symptoms both at baseline and follow-up were randomly selected as the case group, while those without anxiety symptoms both at baseline and follow-up were randomly selected as the control group. The case group and the control group were matched according to age. Finally, 97 cases and 141 controls were included for analysis. Based on the matched case-control study formula, the calculated sample size is 26 for each group when investigating the association between childhood maltreatment and anxiety symptoms, including the parameters in the formula based on previously known parameters: alpha value = 0.05, beta value = 0.20, P_0_ = 0.176 (the exposure rate of suffering childhood maltreatment in the control group, OR = 5.67) (27). However, not sufficient information is provided in previous published articles when investigating the association between *FKBP5* methylation and anxiety symptoms among adolescents. To sum up, the sample size used in the current nested case-control study was 238, which was slightly larger than that used in previous studies focusing on the associations of *FKBP5* methylation with mental disorders (the sample sizes for Han was 202; for Bustamante was 112) (28, 29).

After the procedure had been fully informed in detail, written informed consent was obtained from one of the student's parents or legal guardians and each participating student. The study was performed in accordance with the Declaration of Helsinki, and obtained ethical approval from Sun Yat-sen University School of Public Health Institutional Review Board (Ethics Number: L2017060).

### Measures

#### Anxiety Symptoms

The Generalized Anxiety Disorder Screener (GAD-7) is a 7-item self-report scale designed to assess anxiety symptoms (30). The Chinese version has been well validated and shows good internal consistency (the total Cronbach's alpha = 0.84) in Chinses studies (31, 32). The participants were asked to report the frequency of 7 symptoms of anxiety on a 4-point scale ranging from “not at all” (=0) to “almost every day” (=3), and higher scores indicated more severe anxiety symptoms (31). In our current study, we considered scores 5 as a cutoff score, and those with scores more than 5 were categorized as having anxiety symptoms (30). Students with anxiety symptoms both at baseline and follow-up were considered as the case group, while those without anxiety symptoms both at baseline and follow-up were considered as the control group.

#### Childhood Maltreatment

The experience of childhood maltreatment was measured by the short form of the Childhood Trauma Questionnaire (CTQ-SF) (33, 34). The scale was composed of five subscales (physical abuse, emotional abuse, sexual abuse, physical neglect, and emotional neglect), and each subscale consisted of five questions about history that happened in childhood. The Chinese version of this scale has been validated, and the reliability (the total Cronbach's alpha = 0.73) is also satisfactory in Chinese studies (35). Respondents were asked to report the frequency of these questions on a 5-point scale as follows: never = 1, rarely = 2, sometimes = 3, often = 4, and very often = 5, with a maximum score of 25 of each subscale. In the present study, participants with higher scores indicated that they had experienced more severe maltreatment (36).

#### DNA Methylation

We selected the CpG island in the promoter region of the *FKBP5* gene as the target for methylation analysis. The sequences of CpG islands (chr6: 35728998-35729370 and chr6: 35729453-35729707) were determined through the CpG Island Online Prediction website (http://www.ebi.ac.uk/Tools/seqstats/emboss_cpgplot/) based on the CpG island determination criteria (%GC > 50, length > 200 bp, Obs/Exp CpG > 0.6). Agena EpiDesigner software was used to design PCR primers for the target sequence, and two optimal primer design schemes (#12 and #47, details in Supplementary Material) were selected.

According to the manufacturer's instructions, genomic DNA isolated from peripheral blood was converted to bisulfite by EZ DNA Methylation Kit (Zymo Research, Orange, USA). PCR amplification was performed using the previously designed primers to obtain an amplification product with a T7 RNA polymerase promoter sequence. In the *in-vitro* transcription system, the amplification product was transcribed into RNA fragments by T7 RNA polymerase, followed by base-specific cleavage using RNase A. The resulting small RNA fragments carrying CpG sites differed in their nucleotide composition depending on the bisulfite treatment. DNA methylation levels were detected for each fragment using MassArray EpiTYPER software. The method produces quantitative results for each unit of analysis, called a CpG unit, containing an individual CpG site or an aggregate of several CpG sites whose positions were relatively close. Finally, a total of 71 CpG sites were divided into 42 CpG units (The locations of the CpG units were shown in Table S1).

After the data were obtained, further quality control was performed, including excluding CpG units with <80% of available methylation data to ensure that spurious data were not analyzed (37). Moreover, significantly deviating data points were also excluded (38). A total of 22 CpG units encompassing 40 CpG sites were ultimately qualified for analysis.

#### Covariates

The covariates included age, gender (1 = boy, 2 = girl), body mass index (BMI), family structure, household socioeconomic status (HSS), academic pressure, classmate relationships, teacher-classmate relationships, and current alcohol consumption, which had been reported to be associated with anxiety symptoms in previous studies (39, 40). Family structure was measured by asking whom they lived with at home (the answer was coded as 1 = living with parents, 2 = living with a single parent, and 3 = living with others.). HSS was measured by the respondent's perception of their family socioeconomic status (the answer was coded as 1 = excellent, 2 = good, and 3 = fair or poor). Academic pressure was evaluated by asking students to rate their schoolwork pressure (the answer was coded as 1 = none, 2 = moderate, and 3 = severe). Classmate relationships and teacher-classmate relationships were assessed based on the student's self-rating the quality of their relationships with their classmates and teachers (the answer was coded as 1 = good, 2 = average, and 3 = poor). Current alcohol consumption was assessed by asking the question: “How many days did you drink alcohol during the last 30 days,” and students who responded one or more days were categorized as current drinking (41).

### Statistical Analysis

The current study analyzed data from baseline and follow-up 1 year later. First, descriptive analyses stratified by case and control groups were performed to describe the sample characteristics, and the data were expressed as number (%) and mean (standard deviation, SD). A Chi-square test for categorical variables and a *t*-test for continuous variables were used to compare the differences between the two groups. Second, the variables with *P* < 0.10 in the univariable analysis or widely reported in the previous literature were incorporated into the multiple linear regression models to test the independent association of childhood maltreatment with anxiety symptoms at follow-up (42). Third, multiple linear regression models were performed to explore the association between childhood maltreatment and methylation of each CpG unit, and the association between DNA methylation and anxiety symptoms at follow-up. Fourth, multivariable logistic regression models were used to explore the association of *FKBP5* gene methylation with the status of the case and control. All data analyses were performed using R (version 3.6.1), and all statistical tests were two-sided, with a *P*-value <0.05 considered statistically significant. To address the concern of multiple hypothesis testing and potential Type I errors, the false discovery rate (FDR) was calculated (43). The term of FDR-adjusted *P* was indicated by “*q*,” and the results were considered as nominally significant when *q* < 0.10 (44).

## Result

### Baseline Sample Characteristics

The sample characteristics at baseline are shown in Table 1. The case group (*n* = 97) and the control group (*n* = 141) were matched according to age (*P* = 0.111). The mean (SD) age of the cases and controls were 13.8 (1.5) and 13.5 (1.4) years, respectively. There was a gender difference between the case and control groups. In the case group, 64.9% were female, and the proportion was higher than that in the control group (64.9 vs. 46.1%, *P* = 0.004). The mean scores of each subscale of CTQ in case group were higher than that in control group (physical abuse: 8.1 vs. 5.9; emotional abuse: 12.4 vs. 7.1; sexual abuse: 6.1 vs. 5.2; physical neglect: 8.1 vs. 6.7; emotional neglect: 11.2 vs. 7.0; all *P* < 0.05). There were significant differences between the two groups in gender, living arrangement, HSS, academic pressure, classmate relationships, teacher-classmate relationships, and current drinking (*P* < 0.05).

**Table 1:** Baseline sample characteristics among 238 adolescents.

		**Anxiety symptom**		
**Variable**	**Total, *N* (%)**	**Cases, *n* (%)**	**Controls, *n* (%)**	P*-value
Total	238 (100)	97 (40.8)	141 (59.2)	
Age, mean (SD)	13.6 (1.4)	13.8 (1.5)	13.5 (1.4)	0.111
BMI, mean (SD)	19.6 (3.5)	20.0 (3.6)	19.3 (3.4)	0.124
Gender
Male	110 (46.2)	34 (35.1)	76 (53.9)	**0.004**
Female	128 (53.8)	63 (64.9)	65 (46.1)	
Living arrangement
Living with parents	189 (79.7)	68 (70.1)	121 (86.4)	**0.008**
Living with a single parent	31 (13.1)	18 (18.6)	13 (9.3)	
Living with others	17 (7.2)	11 (11.3)	6 (4.3)	
Missing data	1	NA	NA	
HSS
Excellent	123 (51.7)	41 (42.3)	82 (58.2)	**0.045** ^**a**^
Good	106 (44.5)	51 (52.6)	55 (39.0)	
Fair or poor	9 (3.8)	5 (5.2)	4 (2.8)	
Academic pressure
None	65 (27.4)	7 (7.2)	58 (41.4)	**<0.001**
Moderate	94 (39.7)	29 (29.9)	65 (46.4)	
Severe	78 (32.9)	61 (62.9)	17 (12.1)	
Missing data	1	NA	NA	
Classmate relationships
Good	189 (79.7)	61 (62.9)	128 (91.4)	**<0.001**
Average	35 (14.8)	26 (26.8)	9 (6.4)	
Poor	13 (5.5)	10 (10.3)	3 (2.1)	
Missing data	1	NA	NA	
Teacher-classmate relationships
Good	191 (80.3)	60 (61.9)	131 (92.9)	**<0.001** ^**a**^
Average	45 (18.9)	35 (36.1)	10 (7.1)	
Poor	2 (0.8)	2 (2.1)	0 (0)	
Current drinking				
No	201 (85.9)	74 (78.7)	127 (90.7)	**0.010**
Yes	33 (14.1)	20 (21.3)	13 (9.3)	
Missing data	4	NA	NA	
CTQ scores of physical abuse, mean (SD)	6.8 (2.8)	8.1 (3.6)	5.9 (1.7)	**<0.001**
CTQ scores of emotional abuse, mean (SD)	9.2 (5.1)	12.4 (5.0)	7.1 (3.8)	**<0.001**
CTQ scores of sexual abuse, mean (SD)	5.6 (1.6)	6.1 (2.3)	5.2 (0.8)	**<0.001**
CTQ scores of physical neglect, mean (SD)	7.3 (3.0)	8.1 (3.6)	6.7 (2.4)	**0.001**
CTQ scores of emotional neglect, mean (SD)	8.7 (5.3)	11.2 (5.7)	7.0 (4.2)	**<0.001**
GAD scores at baseline, mean (SD)	6.3 (6.9)	13.1 (4.6)	1.7 (3.5)	**<0.001**
GAD scores at follow-up, mean (SD)	5.5 (6.6)	12.0 (5.2)	1.0 (2.1)	**<0.001**

HSS, household socioeconomic status; NA, not applicable or no data available; *t-tests were used for continuous variables; chi-square tests were used for categorical variables; ^a^Fisher exact tests were used for HSS and teacher-classmate relationships. The bold values represents that the P-value <0.05.

### Association Between Childhood Maltreatment and Anxiety Symptoms at Follow-Up

Overall, the mean (SD) GAD-7 scores of adolescents with and without anxiety symptoms at follow-up were 12.0 (5.2) and 1.0 (2.1), respectively. As presented in Table 2, without adjusting for variables, physical abuse, emotional abuse, sexual abuse, physical neglect, and emotional neglect were significantly associated with anxiety symptoms at follow-up (*P* < 0.05), respectively. However, after adjusting for age, gender, BMI, living arrangement, HSS, academic pressure, classmate relationships, teacher-classmate relationships, current drinking and anxiety symptoms (GAD-7 scores) at baseline, only emotional abuse (β = 0.146, 95% CI = 0.010~0.283) were significantly associated with anxiety symptoms at follow-up.

**Table 2:** Association of childhood maltreatment with anxiety symptoms at follow-up.

	**anxiety symptoms at follow-up (GAD-7 scores)**			
	**Model 1**		**Model 2**	
**Variable**	**β (95% *CI*)**	***P-*value**	**β (95% *CI*)**	***P-*value**
physical abuse (1-score increase)	0.901 (0.629~1.172)	**<0.001**	0.185 (-0.031~0.400)	0.093
emotional abuse (1-score increase)	0.681 (0.539~0.822)	**<0.001**	0.146 (0.010~0.283)	**0.036**
sexual abuse (1-score increase)	0.888 (0.390~1.387)	**0.001**	−0.064 (-0.437~0.309)	0.736
physical neglect (1-score increase)	0.507 (0.236~0.778)	**<0.001**	−0.020 (-0.216~0.177)	0.844
emotional neglect (1-score increase)	0.512 (0.367~0.657)	**<0.001**	0.087 (-0.036~0.210)	0.164

*The GAD scores of anxiety symptom at follow-up was incorporated into the linear regression model as a dependent variable; Model 1was unadjusted linear regression models; Model 2 adjusted for age, gender, BMI, living arrangement, HSS, academic pressure, classmate relationships, teacher-classmate relationships, current drinking and anxiety symptoms at baseline. The bold values represents that the P-value <0.05*.

### Association of Childhood Maltreatment With DNA Methylation in *FKBP5* Gene

After adjusting for covariates, our final multiple linear regression model showed that childhood maltreatment was negatively associated with DNA methylation levels at most CpG units within the *FKBP5* gene promoter region. Physical and emotional neglect was associated with an increased risk of hypomethylation at most CpG units (*P* < 0.05). Furthermore, students who experienced emotional abuse were more likely to get a higher methylation level at *FKBP5*-47 CpG 30.31.32 (*P* < 0.05). After further correction for multiple testing, neither emotional abuse, physical neglect, nor emotional neglect showed an association with DNA methylation in the *FKBP5* gene (*q* > 0.10). The associations of all types of childhood maltreatment with DNA methylation in the *FKBP5* gene promoter region were presented in Table S2.

### Association of DNA Methylation in FKBP5 Gene With Anxiety Symptoms at Follow-Up

As shown in Table 3, among the 26 CpG units in the promoter region of the *FKBP5* gene detected in our study, the DNA methylation level at *FKBP5*-12 CpG 15 (β = −0.263, *P* < 0.05) was associated with an increased risk of anxiety symptoms after adjusting for age, gender, BMI, living arrangement, HSS, academic pressure, classmate relationships, teacher-classmate relationships, current drinking, and anxiety symptoms at baseline. However, after further correction for multiple testing, CpG 15 did not show an association with anxiety symptoms (*q* > 0.10). As shown in Table S3, the multivariable logistic regression models showed that the DNA methylation level at *FKBP5*-12 CpG 15 and at *FKBP5*-47 CpG 37 was associated with the status of the case (*P* < 0.05) after adjusting for age, gender, BMI, living arrangement, HSS, academic pressure, classmate relationships, teacher-classmate relationships, current drinking. However, after further correction for multiple testing, *FKBP5*-12 CpG 15 and *FKBP5*-47 CpG 37 did not show associations with the status of the case (*q* > 0.10).

**Table 3:** Association of DNA methylation in *FKBP5* gene with anxiety symptoms at follow-up.

	**Methylation level**	Anxiety symptoms at follow-up*		
**CPG unit**	**(mean ±SD)**	**β (95% *CI*)**	***P-*value**	***q-*value**
*FKBP5*-12 CpG 1	0.39 ± 0.68	−0.675 (-1.559~0.208)	0.133	0.659
*FKBP5*-12 CpG 2	2.81 ± 2.01	0.168 (-0.113~0.450)	0.240	0.659
*FKBP5*-12 CpG 3	2.35 ± 2.43	−0.089 (-0.325~0.147)	0.460	0.943
*FKBP5*-12 CpG 5.6.7	40.45 ± 14.92	−0.004 (-0.041~0.033)	0.830	0.943
*FKBP5*-12 CpG 8	4.73 ± 4.36	−0.012 (-0.141~0.117)	0.859	0.943
*FKBP5*-12 CpG 9	4.81 ± 4.29	−0.031 (-0.163~0.102)	0.646	0.943
*FKBP5*-12 CpG 10.11	2.96 ± 1.11	−0.055 (-0.565~0.456)	0.833	0.943
*FKBP5*-12 CpG 12	16.26 ± 12.83	0.031 (-0.017~0.079)	0.207	0.659
*FKBP5*-12 CpG 13	53.40 ± 20.86	−0.002 (-0.028~0.024)	0.901	0.943
*FKBP5*-12 CpG 14	2.80 ± 1.90	0.272 (-0.022~0.566)	0.070	0.659
*FKBP5*-12 CpG 15	2.25 ± 2.78	−0.263 (-0.458~-0.069)	**0.008**	0.180
*FKBP5*-12 CpG 17.18.19	12.62 ± 7.04	−0.017 (-0.098~0.064)	0.680	0.943
*FKBP5*-47 CpG 2.3	2.43 ± 2.94	0.037 (-0.164~0.238)	0.715	0.943
*FKBP5*-47 CpG 4.5.6.7	2.95 ± 2.55	0.162 (-0.066~0.390)	0.163	0.659
*FKBP5*-47 CpG 8.9	10.79 ± 8.47	0.001 (-0.069~0.072)	0.969	0.969
*FKBP5*-47 CpG 13.14.15	1.21 ± 1.39	−0.161 (-0.589~0.266)	0.457	0.943
*FKBP5*-47 CpG 16.17	2.02 ± 2.87	0.013 (-0.188~0.215)	0.897	0.943
*FKBP5*-47 CpG 25.26	1.38 ± 2.09	−0.085 (-0.375~0.204)	0.562	0.943
*FKBP5*-47 CpG 30.31.32	3.59 ± 3.03	0.117 (-0.077~0.310)	0.235	0.659
*FKBP5*-47 CpG 33.34.35	14.79 ± 6.31	0.023 (-0.068~0.114)	0.622	0.943
*FKBP5*-47 CpG 37	1.49 ± 2.19	−0.191 (-0.461~0.079)	0.165	0.659
*FKBP5*-47 CpG 51	11.60 ± 5.77	−0.013 (-0.113~0.086)	0.789	0.943

FKBP5-12 and FKBP5-47 represent DNA fragment position corresponding to primer #12 and #47, respectively; The GAD scores of anxiety symptom at follow-up were incorporated into the linear regression model as a dependent variable. *The multiple linear regression models for anxiety symptoms at follow-up were adjusted for age, gender, BMI, living arrangement, HSS, academic pressure, classmate relationships, teacher-classmate relationships, current drinking and anxiety symptoms at baseline. The bold values represents that the P-value <0.05.

## Discussion

Our study indicated that there were significant differences between adolescents with and without anxiety symptoms in living arrangements, HSS, academic pressure, classmate relationships, teacher-classmate relationships, and current drinking. The confounding effects of these variables need to be considered when exploring the association between childhood maltreatment and anxiety symptoms. Besides, these results may help us identify adolescents at a higher risk of developing anxiety symptoms.

The current study provided evidence that childhood emotional abuse was associated with anxiety symptoms at follow-up after adjusting for age, gender, BMI, living arrangement, HSS, academic pressure, classmate relationships, teacher-classmate relationships, current drinking, and anxiety symptoms at baseline, which was consistent with previous studies. Banducci et al. (45) reported that individuals exposed to childhood emotional abuse were more likely to have a higher level of anxiety symptoms in adulthood. In a cross-sectional study in Finland, we could observe that severe emotional abuse was an important predictor of anxiety symptoms in adulthood (46). Similarly, a longitudinal study in the Netherlands suggested that people with emotional abuse in childhood were more likely to develop more unfavorable personality characteristics (such as neuroticism and hopelessness) and adopt poorer coping styles, which were negatively associated with the remission of anxiety symptoms (47). The experience of childhood abuse disturbed the development of individual emotional regulation. People who had been abused were lacking in emotional support, and were challenging to alleviate their negative emotions, which eventually led to the onset of anxiety symptoms and even developed into anxiety disorders subsequently (11, 48). Another explanation from the neurobiological mechanism was that the experience of childhood maltreatment could influence the normal function of the HPA axis. Sustained exposure to adverse life events such as childhood maltreatment may leave the stress response system in a hypersensitive state. To adapt to such changes, the organism will elevate the secretion of corticotropin-releasing hormone (CRH), leading to the hyperreactivity of the HPA axis as well as the autonomic nervous system (24, 49). The dysfunction of the HPA axis has been shown to be associated with an increased risk of poor mental outcomes (50).

Furthermore, one significant finding of this study was that a history of childhood physical and emotional neglect was negatively associated with DNA methylation in the promoter region of the *FKBP5* gene after adjusting for the covariates. Although the association did not withstand the multiple hypotheses, compelling evidence suggested that individuals who were exposed to physical and emotional neglect were more likely to get a lower methylation level of *FKBP5* DNA methylation. Klengel and colleagues demonstrated that individuals with childhood abuse exhibited significant demethylation of intron 7 of the *FKBP5* gene compared to those without abuse (21). Moreover, a prospective longitudinal study reported that early life stress, especially childhood abuse, was a significant predictor of altered DNA methylation of an intron of the *FKBP5* gene (49). Considering that childhood maltreatment occurred during a critical period of brain development when brain plasticity was enhanced, children were vulnerable to the neuropsychological effects of adverse life events, resulting in an increased likelihood of epigenomic alterations (51). *FKBP5* gene plays a vital role in the stress response system, and childhood maltreatment as an environmental stressor may be more likely to cause alterations in *FKBP5* gene methylation, and the alteration in DNA methylation of the *FKBP5* gene has been shown to be a risk factor for mental disorders (22, 24, 49). These findings recommended the necessity for schools and families to be aware of the dangers of childhood maltreatment and provide emotional support to adolescents who have experienced childhood maltreatment.

Additionally, our final multiple linear regression models showed that DNA methylation of the promoter region of the *FKBP5* gene showed an association with anxiety symptoms at follow-up after adjusting for the covariates. However, the association did not withstand the multiple hypothesis testing, consistent with previous literature. In the Detroit Neighborhood Health Study, Bustamante et al. reported that DNA methylation in the promoter region of the *FKBP5* gene was not a significant predictor of mental health (29). However, extensive studies have highlighted the importance of DNA methylation of the *FKBP5* gene in predicting mental health outcomes (44, 51). FKBP5 plays an important role in the intracellular ultra-short negative feedback loop that regulates GR activity. Moreover, demethylation of *FKBP5* increases the expression of *FKBP5*, resulting in diminished negative feedback and reduced sensitivity to GR, which leads to prolonged glucocorticoid exposure and excessive stress response (22, 24). Nevertheless, this study did not confirm that *FKBP5* DNA methylation was associated with anxiety symptoms at follow-up, a plausible explanation was that the effect of methylation of an individual CpG unit was weak, and most CpG units could not survive the multiple hypothesis testing (44), thereby the combined effect should be considered in later studies.

Our study has several limitations. First, we used a retrospective self-report scale to examine childhood maltreatment history, which can not preclude the recall bias. Moreover, adolescents might not be reluctant to respond to the experience of childhood maltreatment, resulting in an underestimate of maltreatment. Second, our study sample only derived from adolescents in school on the day of the survey and did not include those who were absent from school. The experience of childhood maltreatment and anxiety symptoms might be more prevalent in those students. Third, we only measured methylation in peripheral blood, and it was not clear whether the result accurately reflected DNA methylation in the brain. However, using peripheral blood is a widely accepted method in detecting DNA methylation. This study only included methylation of the promoter region of the *FKBP5* gene, and methylation of other regions should be considered in further studies. Fourth, it is possible that our study was underpowered to detect the associations between *FKBP5* methylation and anxiety symptoms. However, a *post-hoc* power calculation using in G^*^Power software indicated that the sample size in the current study was adequately powered (>90%) to detect medium effects sizes. Despite these limitations, the strength of the current study is that our study utilized a prospective nested case-control study design and has a larger sample size than previous studies. The study population is focused on adolescents aged 13–18 years old, and we are concerned with the subclinical state of anxiety disorders, aiming to identify early biomarkers of mental health in adolescents for early intervention.

## Conclusion

In summary, our study demonstrates that childhood emotional abuse could be an important risk factor for subsequent anxiety symptoms; childhood maltreatment is not significantly associated with *FKBP5* DNA methylation; DNA methylation of *FKBP5* gene promotor could not predict the subsequent anxiety symptoms. Based on the current study results, more attention should be paid to the mental health of adolescents with childhood maltreatment. Besides, the establishment of a nationwide surveillance system on adolescent mental health is strongly recommended.

## Data Availability Statement

The original contributions presented in the study are included in the article/Supplementary Material, further inquiries can be directed to the corresponding authors.

## Ethics Statement

The studies involving human participants were reviewed and approved by Sun Yat-sen University School of Public Health Institutional Review Board. Written informed consent to participate in this study was provided by the participants' legal guardian/next of kin.

## Author Contributions

The study was originally conceived and designed by CL and LG. Investigation and data collection were performed by WLa, WLi, XD, YG, and WW. Data analysis was performed by WLa, WLi, and WW. WLa and WLi wrote the first draft of the manuscript, with a number of edits from CL and LG. All authors were in agreement with the final submitted manuscript.

## Funding

This work was supported by the National Natural Science Foundation of China (Grant Nos. 81761128030 and 81903339) and Natural Science Foundation of Guangdong Province (Grant No. 2019A1515011091).

## Conflict of Interest

The authors declare that the research was conducted in the absence of any commercial or financial relationships that could be construed as a potential conflict of interest.

## Publisher's Note

All claims expressed in this article are solely those of the authors and do not necessarily represent those of their affiliated organizations, or those of the publisher, the editors and the reviewers. Any product that may be evaluated in this article, or claim that may be made by its manufacturer, is not guaranteed or endorsed by the publisher.
