# Supplementary material for: Association Between Childhood Maltreatment, FKBP5 Gene Methylation, and Anxiety Symptoms Among Chinese Adolescents: A Nested Case-Control Study
Source: Front Psychiatry. 2022 Feb 3;13:761898. doi: 10.3389/fpsyt.2022.761898 (PMC8850925; doi:10.3389/fpsyt.2022.761898)
Supplement: Supplementary file 1 [file Data_Sheet_1.docx]

Supplement

There is a total of 20 CpG sites in the DNA fragment corresponding to primer #12, only 19 of them are covered in our study.

**Primer #12**

Forward PCR primer:

5’ - aggaagagagGTTGGAGAGGGTTTATTGGGAGT - 3’ (lower case letters indicate T7 primers)

Reverse PCR primer:

5’- cagtaatacgactcactatagggagaaggctCCCCAATTACCTTAACATCAATAAC - 3’ (lower case letters indicate T7 primers)

There is a total of 51 CpG sites in the DNA fragment corresponding to primer #47, only 36 of them are covered in our study.

**Primer #47**

Forward PCR primer:

5’ - aggaagagagGAGATTTTGAGAATTTGGGTTTAG - 3’ (lower case letters indicate T7 primers)

Reverse PCR primer:

5’ - cagtaatacgactcactatagggagaaggctACTTCCCTTACAAAAAACCAAAAAA - 3’

(lower case letters indicate T7 primers)

**Supplemental Table S1.** The location and methylation level of each CpG unit in the *FKBP5* gene.

|  |  | Methylation level |
| --- | --- | --- |
| CPG unit | Location (chr6) | (mean±SD) |
| *FKBP5*-12 CpG 1 | 35729435 | 0.39±0.68 |
| *FKBP5*-12 CpG 2 | 35729454 | 2.81±2.01 |
| *FKBP5*-12 CpG 3 | 35729466 | 2.35±2.43 |
| *FKBP5*-12 CpG 5.6.7 | 35729513 | 40.45±14.92 |
|  | 35729515 |  |
|  | 35729519 |  |
| *FKBP5*-12 CpG 8 | 35729528 | 4.73±4.36 |
| *FKBP5*-12 CpG 9 | 35729536 | 4.81±4.29 |
| *FKBP5*-12 CpG 10.11 | 35729558 | 2.96±1.11 |
|  | 35729561 |  |
| *FKBP5*-12 CpG 12 | 35729568 | 16.26±12.83 |
| *FKBP5*-12 CpG 13 | 35729574 | 53.40±20.86 |
| *FKBP5*-12 CpG 14 | 35729611 | 2.80±1.90 |
| *FKBP5*-12 CpG 15 | 35729617 | 2.25±2.78 |
| *FKBP5*-12 CpG 17.18.19 | 35729658 | 12.62±7.04 |
|  | 35729665 |  |
|  | 35729667 |  |
| *FKBP5*-47 CpG 2.3 | 35728891 | 2.43±2.94 |
|  | 35728901 |  |
| *FKBP5*-47 CpG 4.5.6.7 | 35728911 | 2.95±2.55 |
|  | 35728914 |  |
|  | 35728919 |  |
|  | 35728921 |  |
| *FKBP5*-47 CpG 8.9 | 35728933 | 10.79±8.47 |
|  | 35728937 |  |
| *FKBP5*-47 CpG 13.14.15 | 35728974 | 1.21±1.39 |
|  | 35728977 |  |
|  | 35728980 |  |
|  | 35728984 |  |
| *FKBP5*-47 CpG 16.17 | 35728986 | 2.02±2.87 |
|  | 35728974 |  |
| *FKBP5*-47 CpG 25.26 | 35729064 | 1.38±2.09 |
|  | 35729070 |  |
| *FKBP5*-47 CpG 30.31.32 | 35729106 | 3.59±3.03 |
|  | 35729109 |  |
|  | 35729112 |  |
| *FKBP5*-47 CpG 33.34.35 | 35729118 | 14.79±6.31 |
|  | 35729120 |  |
|  | 35729122 |  |
| *FKBP5*-47 CpG 37 | 35729159 | 1.49±2.19 |
| *FKBP5*-47 CpG 51 | 35729357 | 11.60±5.77 |

**Supplemental Table S2.** Adjusted association of childhood maltreatment with DNA methylation in *FKBP5* gene

|  | Physical abuse (n=238) | | |  | Emotional abuse (n=238) | | |  | Sexual abuse (n=238) | | |  | Physical neglect (n=238) | | |  | Emotional neglect (n=238) | | |
| --- | --- | --- | --- | --- | --- | --- | --- | --- | --- | --- | --- | --- | --- | --- | --- | --- | --- | --- | --- |
| CPG site | *β* | *P** | *q* |  | *β* | *P** | *q* |  | *β* | *P** | *q* |  | *β* | *P** | *q* |  | *β* | *P** | *q* |
| *FKBP5*-12 CpG 1 | 0.026 | 0.150 | 0.741 |  | -0.016 | 0.156 | 0.606 |  | -0.009 | 0.793 | 0.961 |  | 0.003 | 0.878 | 0.962 |  | -0.007 | 0.495 | 0.631 |
| *FKBP5*-12 CpG 2 | -0.009 | 0.873 | 0.933 |  | 0.035 | 0.301 | 0.827 |  | 0.020 | 0.826 | 0.961 |  | 0.078 | 0.113 | 0.355 |  | 0.054 | 0.076 | 0.208 |
| *FKBP5*-12 CpG 3 | 0.070 | 0.276 | 0.741 |  | -0.035 | 0.389 | 0.859 |  | 0.078 | 0.487 | 0.961 |  | -0.127 | **0.033** | 0.338 |  | -0.001 | 0.981 | 0.981 |
| *FKBP5*-12 CpG 5.6.7 | -0.335 | 0.403 | 0.741 |  | -0.037 | 0.884 | 0.926 |  | -0.097 | 0.889 | 0.961 |  | -0.418 | 0.263 | 0.644 |  | -0.409 | 0.068 | 0.208 |
| *FKBP5*-12 CpG 8 | 0.024 | 0.843 | 0.933 |  | 0.104 | 0.162 | 0.606 |  | 0.330 | 0.104 | 0.923 |  | -0.234 | **0.034** | 0.338 |  | -0.108 | 0.107 | 0.262 |
| *FKBP5*-12 CpG 9 | 0.098 | 0.388 | 0.741 |  | -0.019 | 0.790 | 0.883 |  | 0.246 | 0.210 | 0.923 |  | -0.093 | 0.382 | 0.841 |  | -0.071 | 0.268 | 0.540 |
| *FKBP5*-12 CpG 10.11 | 0.027 | 0.339 | 0.741 |  | 0.019 | 0.283 | 0.827 |  | -0.058 | 0.246 | 0.923 |  | 0.001 | 0.962 | 0.962 |  | -0.009 | 0.587 | 0.680 |
| *FKBP5*-12 CpG 12 | -0.264 | 0.495 | 0.779 |  | 0.197 | 0.391 | 0.859 |  | 0.842 | 0.172 | 0.923 |  | 0.270 | 0.438 | 0.875 |  | 0.084 | 0.690 | 0.759 |
| *FKBP5*-12 CpG 13 | -0.597 | 0.288 | 0.741 |  | -0.089 | 0.803 | 0.883 |  | -0.048 | 0.961 | 0.961 |  | -0.869 | 0.093 | 0.351 |  | -0.712 | **0.023** | 0.166 |
| *FKBP5*-12 CpG 14 | 0.018 | 0.746 | 0.919 |  | -0.016 | 0.623 | 0.882 |  | -0.007 | 0.937 | 0.961 |  | -0.030 | 0.546 | 0.961 |  | 0.021 | 0.479 | 0.631 |
| *FKBP5*-12 CpG 15 | -0.083 | 0.337 | 0.741 |  | -0.078 | 0.103 | 0.606 |  | 0.150 | 0.252 | 0.923 |  | -0.004 | 0.957 | 0.962 |  | -0.099 | **0.019** | 0.166 |
| *FKBP5*-12 CpG 17.18.19 | -0.094 | 0.632 | 0.919 |  | 0.068 | 0.584 | 0.882 |  | -0.025 | 0.945 | 0.961 |  | -0.013 | 0.943 | 0.962 |  | 0.092 | 0.407 | 0.604 |
| *FKBP5*-47 CpG 2.3 | 0.113 | 0.168 | 0.741 |  | 0.024 | 0.642 | 0.882 |  | -0.119 | 0.397 | 0.961 |  | 0.008 | 0.915 | 0.962 |  | 0.005 | 0.913 | 0.956 |
| *FKBP5*-47 CpG 4.5.6.7 | 0.061 | 0.434 | 0.741 |  | 0.002 | 0.961 | 0.961 |  | -0.120 | 0.323 | 0.961 |  | -0.122 | 0.061 | 0.338 |  | -0.082 | **0.038** | 0.166 |
| *FKBP5*-47 CpG 8.9 | -0.022 | 0.933 | 0.933 |  | -0.208 | 0.165 | 0.606 |  | 0.056 | 0.895 | 0.961 |  | -0.410 | 0.061 | 0.338 |  | -0.249 | 0.055 | 0.203 |
| *FKBP5*-47 CpG 13.14.15 | -0.013 | 0.752 | 0.919 |  | -0.053 | **0.043** | 0.478 |  | -0.090 | 0.178 | 0.923 |  | 0.013 | 0.718 | 0.962 |  | -0.021 | 0.349 | 0.591 |
| *FKBP5*-47 CpG 16.17 | -0.007 | 0.927 | 0.933 |  | 0.031 | 0.545 | 0.882 |  | -0.063 | 0.647 | 0.961 |  | 0.004 | 0.961 | 0.962 |  | 0.050 | 0.27 | 0.540 |
| *FKBP5*-47 CpG 25.26 | 0.032 | 0.680 | 0.919 |  | -0.012 | 0.775 | 0.883 |  | 0.023 | 0.829 | 0.961 |  | 0.033 | 0.568 | 0.961 |  | 0.022 | 0.516 | 0.631 |
| *FKBP5*-47 CpG 30.31.32 | -0.069 | 0.404 | 0.741 |  | 0.108 | **0.036** | 0.478 |  | -0.036 | 0.800 | 0.961 |  | 0.030 | 0.699 | 0.962 |  | 0.038 | 0.412 | 0.604 |
| *FKBP5*-47 CpG 33.34.35 | 0.257 | 0.118 | 0.741 |  | 0.060 | 0.556 | 0.882 |  | 0.108 | 0.703 | 0.961 |  | -0.252 | 0.096 | 0.351 |  | -0.190 | **0.036** | 0.166 |
| *FKBP5*-47 CpG 37 | -0.086 | 0.166 | 0.741 |  | 0.013 | 0.724 | 0.883 |  | -0.086 | 0.395 | 0.961 |  | 0.021 | 0.700 | 0.962 |  | 0.034 | 0.297 | 0.544 |
| *FKBP5*-47 CpG 51 | -0.118 | 0.438 | 0.741 |  | -0.055 | 0.564 | 0.882 |  | 0.171 | 0.511 | 0.961 |  | -0.159 | 0.256 | 0.644 |  | -0.206 | **0.015** | 0.166 |

Note: *FKBP5*-12 and *FKBP5*-47 represent DNA fragment position corresponding to primer #12 and #47, respectively;

* The multiple linear regression models were adjusted for age, gender, BMI, living arrangement, HSS, academic pressure, classmate relationships, teacher-classmate relationships and current drinking;

**Supplemental Table S3.** Adjusted association of DNA methylation in the *FKBP5* gene with the status of the case.

|  | The status of the case* | | |
| --- | --- | --- | --- |
| CPG unit | *OR* (95% *CI*) | *P* | *q* |
| *FKBP5*-12 CpG 1 | 0.73 (0.42-1.26) | 0.263 | 0.742 |
| *FKBP5*-12 CpG 2 | 0.98 (0.81-1.18) | 0.819 | 0.929 |
| *FKBP5*-12 CpG 3 | 0.96 (0.82-1.12) | 0.617 | 0.929 |
| *FKBP5*-12 CpG 5.6.7 | 1.00 (0.98-1.03) | 0.819 | 0.929 |
| *FKBP5*-12 CpG 8 | 1.05 (0.97-1.14) | 0.215 | 0.742 |
| *FKBP5*-12 CpG 9 | 1.02 (0.93-1.11) | 0.658 | 0.929 |
| *FKBP5*-12 CpG 10.11 | 0.89 (0.63-1.24) | 0.482 | 0.929 |
| *FKBP5*-12 CpG 12 | 1.00 (0.97-1.03) | 0.868 | 0.929 |
| *FKBP5*-12 CpG 13 | 1.00 (0.98-1.02) | 0.850 | 0.929 |
| *FKBP5*-12 CpG 14 | 0.96 (0.79-1.16) | 0.658 | 0.929 |
| *FKBP5*-12 CpG 15 | 0.87 (0.76-0.99) | **0.042** | 0.383 |
| *FKBP5*-12 CpG 17.18.19 | 0.95 (0.90-1.00) | 0.052 | 0.383 |
| *FKBP5*-47 CpG 2.3 | 1.01 (0.89-1.15) | 0.860 | 0.929 |
| *FKBP5*-47 CpG 4.5.6.7 | 1.08 (0.94-1.25) | 0.279 | 0.742 |
| *FKBP5*-47 CpG 8.9 | 1.00 (0.96-1.04) | 0.955 | 0.955 |
| *FKBP5*-47 CpG 13.14.15 | 0.84 (0.62-1.11) | 0.236 | 0.742 |
| *FKBP5*-47 CpG 16.17 | 0.94 (0.81-1.08) | 0.382 | 0.840 |
| *FKBP5*-47 CpG 25.26 | 1.17 (0.96-1.43) | 0.117 | 0.643 |
| *FKBP5*-47 CpG 30.31.32 | 0.99 (0.87-1.12) | 0.887 | 0.929 |
| *FKBP5*-47 CpG 33.34.35 | 0.97 (0.91-1.03) | 0.304 | 0.742 |
| *FKBP5*-47 CpG 37 | 0.84 (0.70-0.99) | **0.044** | 0.383 |
| *FKBP5*-47 CpG 51 | 0.99 (0.92-1.05) | 0.671 | 0.929 |

Note: *FKBP5*-12 and *FKBP5*-47 represent DNA fragment position corresponding to primer #12 and #47, respectively; The GAD scores of anxiety symptom at follow-up were incorporated into the linear regression model as a dependent variable.

* The multivariable logistic regression models were adjusted for age, gender, BMI, living arrangement, HSS, academic pressure, classmate relationships, teacher-classmate relationships, and current drinking.
